# {2,2′-[4,5-Dibromo-*o*-phenyl­enebis(nitrilo­dimethyl­idyne)]diphenolato-κ^4^ *O*,*N*,*N*′,*O*′}(methanol-κ*O*)copper(II)

**DOI:** 10.1107/S1600536809011179

**Published:** 2009-03-31

**Authors:** Jianxin Xing

**Affiliations:** a Department of Biology, Dezhou University, Dezhou 253023, People’s Republic of China

## Abstract

In the title compound, [Cu(C_20_H_12_Br_2_N_2_O_2_)(CH_3_OH)], the Cu^II^ ion, and the C, O and hydr­oxy H atoms of the coordinated methanol mol­ecule are located on a twofold rotation axis, while the methyl H atoms are disordered over two sites about the rotation axis. The Cu^II^ ion is coordinated by two N atoms [Cu—N = 1.960 (4) Å] and two O atoms [Cu—O = 1.908 (4) Å] from the tetra­dentate Schiff base ligand and by one O atom [Cu—O = 2.324 (6) Å] of the methanol molecule in a square-pyramidal geometry. In the crystal structure, inter­molecular O—H⋯O hydrogen bonds link complex mol­ecules into extended chains along [001].

## Related literature

For a related crystal structure, see Saha *et al.* (2007 ▶). For general background related to Schiff base compounds, see: Ghosh *et al.* (2006 ▶); Nayka *et al.* (2006 ▶); Singh *et al.* (2007 ▶); Yu *et al.* (2007 ▶).
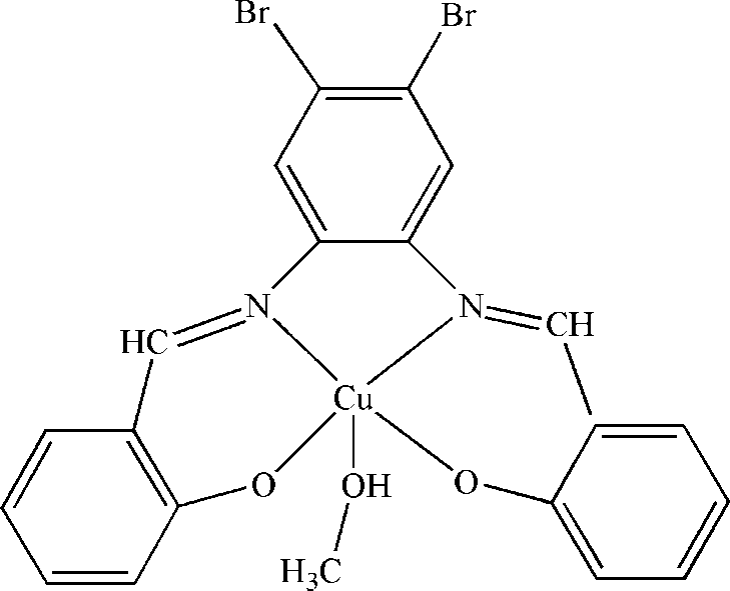


## Experimental

### 

#### Crystal data

[Cu(C_20_H_12_Br_2_N_2_O_2_)(CH_4_O)]
*M* *_r_* = 567.72
Orthorhombic, 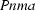

*a* = 19.164 (4) Å
*b* = 19.416 (4) Å
*c* = 5.3287 (10) Å
*V* = 1982.7 (6) Å^3^
*Z* = 4
Mo *K*α radiation
μ = 5.16 mm^−1^
*T* = 273 K
0.21 × 0.15 × 0.13 mm

#### Data collection

Bruker APEXII CCD area-detector diffractometer
Absorption correction: multi-scan (*SADABS*; Sheldrick, 1996 ▶) *T*_min_ = 0.411, *T*_max_ = 0.554 (expected range = 0.379–0.511)
9881 measured reflections
2004 independent reflections
1517 reflections with *I* > 2σ(*I*)
*R*_int_ = 0.046

#### Refinement

*R*[*F* ^2^ > 2σ(*F* ^2^)] = 0.043
*wR*(*F* ^2^) = 0.111
*S* = 1.06
2004 reflections
137 parameters
242 restraints
H-atom parameters constrained
Δρ_max_ = 1.23 e Å^−3^
Δρ_min_ = −1.82 e Å^−3^

### 

Data collection: *APEX2* (Bruker, 2004 ▶); cell refinement: *SAINT-Plus* (Bruker, 2001 ▶); data reduction: *SAINT-Plus*; program(s) used to solve structure: *SHELXS97* (Sheldrick, 2008 ▶); program(s) used to refine structure: *SHELXL97* (Sheldrick, 2008 ▶); molecular graphics: *SHELXTL* (Sheldrick, 2008 ▶); software used to prepare material for publication: *SHELXTL*.

## Supplementary Material

Crystal structure: contains datablocks global, I. DOI: 10.1107/S1600536809011179/lh2794sup1.cif

Structure factors: contains datablocks I. DOI: 10.1107/S1600536809011179/lh2794Isup2.hkl

Additional supplementary materials:  crystallographic information; 3D view; checkCIF report

## Figures and Tables

**Table 1:** Hydrogen-bond geometry (Å, °)

*D*—H⋯*A*	*D*—H	H⋯*A*	*D*⋯*A*	*D*—H⋯*A*
O2—H2*A*⋯O1^i^	0.82	2.30	3.009 (6)	145

Symmetry code: (i) 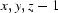.

**Fig. 1.**
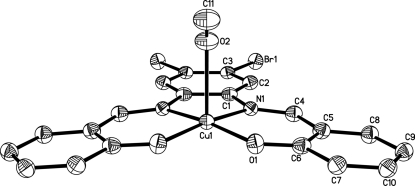
The molecular structure of the title compound with the atom-labelling scheme. Displacement ellipsoids are drawn at the 30% probability level. All H-atoms are omitted for clarity. Unlabelled atoms are related by the symmetry operator (x, -y+3/2, z).

## supplementary crystallographic information

### Comment

Schiff-bases have played an important role in the development of coordination chemistry as they readily form stable complexes with most of the transition metals, in which some may exhibit interesting properties (Yu *et al.*, 2007; Ghosh *et al.*, 2006; Singh *et al.*, 2007; Nayka *et al.*, 2006). Here, we report a new Cu(II) complex based on the tetradentate Schiff-base ligand 4,5-dibromo-1,2-diaminobenzene-*N*,*N*'-bis (salicylideneimine).

The molecular structure of the title compound is shown in Fig. 1. The Cu^II^ ion is pentacoordinated with the four basal sites occupied by two N atoms and two O atoms of the Schiff-base ligand,  while the apical site is occupied by the O atom of the coordinated methanol molecule. The Cu^II^ ion is displaced towards the Cu—O_methanol_ bond from the plane formed by the two N atoms and two O atoms by 0.1017 Å,. The coordination geometry of the Cu^II^ ion is square-pyramidal. The Cu—N, Cu—O and Cu—O_methanol_ bond lengths are consistent with the corresponding distances in aqua-(*N*,*N*'-ethylenebis(3-methoxysalicylaldiminato)-N,*N*',\ *O*,*O*')copper(II) (Saha, *et al.*, 2007).

### Experimental

The Schiff base ligand was synthesized by condensation of 4,5-dibromo-1,2-diaminobenzene and 2-hydroxy-benzaldehyde with the ratio 1:2 in ethanol. The synthesis of the title complex was carried out by reacting Cu(ClO_4_)_2_.6H_2_O, and the schiff-base ligand (1:1, molar ratio) in methanol. After the stirring process was continued for about 10 min at room temperature, the mixture was filtered and the filtrate was allowed to partial evaporate in air for sevral days to produce crystals suitable for X-ray diffraction with a yield about 55%.

### Refinement

H atoms were included using the HFIX command in *SHELXL-97* (Sheldrick, 2008), with C—H = 0.96 and 0.93 Å; O-H = 0.82Å and were allowed for as riding atoms with *U*_iso_(H) = 1.5*U*_eq_(C_methyl_) and (*U*_iso_(H) = 1.2*U*_eq_(C,O).

### Crystal data

**Table:**

[Cu(C_20_H_12_Br_2_N_2_O_2_)(CH_4_O)]	*F*(000) = 1116
*M**_r_* = 567.72	*D*_x_ = 1.902 Mg m^−^^3^*D*_m_ = 1.902 Mg m^−^^3^*D*_m_ measured by not measured
Orthorhombic, *P**n**m**a*	Mo *K*α radiation, λ = 0.71073 Å
Hall symbol: -P 2ac 2n	Cell parameters from 1834 reflections
*a* = 19.164 (4) Å	θ = 3.0–22.2°
*b* = 19.416 (4) Å	µ = 5.16 mm^−^^1^
*c* = 5.3287 (10) Å	*T* = 273 K
*V* = 1982.7 (6) Å^3^	Block, red
*Z* = 4	0.21 × 0.15 × 0.13 mm

### Data collection

**Table:**

Bruker APEXII CCD area-detector diffractometer	2004 independent reflections
Radiation source: fine-focus sealed tube	1517 reflections with *I* > 2σ(*I*)
graphite	*R*_int_ = 0.046
φ and ω scans	θ_max_ = 26.0°, θ_min_ = 2.1°
Absorption correction: multi-scan (*SADABS*; Sheldrick, 1996)	*h* = −23→16
*T*_min_ = 0.411, *T*_max_ = 0.554	*k* = −23→23
9881 measured reflections	*l* = −6→6

### Refinement

**Table:**

Refinement on *F*^2^	Primary atom site location: structure-invariant direct methods
Least-squares matrix: full	Secondary atom site location: difference Fourier map
*R*[*F*^2^ > 2σ(*F*^2^)] = 0.043	Hydrogen site location: inferred from neighbouring sites
*wR*(*F*^2^) = 0.111	H-atom parameters constrained
*S* = 1.06	*w* = 1/[σ^2^(*F*_o_^2^) + (0.0453*P*)^2^ + 5.5878*P*] where *P* = (*F*_o_^2^ + 2*F*_c_^2^)/3
2004 reflections	(Δ/σ)_max_ < 0.001
137 parameters	Δρ_max_ = 1.23 e Å^−^^3^
242 restraints	Δρ_min_ = −1.82 e Å^−^^3^

### Special details

**Table:**

Geometry. All e.s.d.'s (except the e.s.d. in the dihedral angle between two l.s. planes) are estimated using the full covariance matrix. The cell e.s.d.'s are taken into account individually in the estimation of e.s.d.'s in distances, angles and torsion angles; correlations between e.s.d.'s in cell parameters are only used when they are defined by crystal symmetry. An approximate (isotropic) treatment of cell e.s.d.'s is used for estimating e.s.d.'s involving l.s. planes.
Refinement. Refinement of *F*^2^ against ALL reflections. The weighted *R*-factor *wR* and goodness of fit *S* are based on *F*^2^, conventional *R*-factors *R* are based on *F*, with *F* set to zero for negative *F*^2^. The threshold expression of *F*^2^ > σ(*F*^2^) is used only for calculating *R*-factors(gt) *etc*. and is not relevant to the choice of reflections for refinement. *R*-factors based on *F*^2^ are statistically about twice as large as those based on *F*, and *R*- factors based on ALL data will be even larger.

### Fractional atomic coordinates and isotropic or equivalent isotropic displacement parameters (Å^2^)

**Table:**

	*x*	*y*	*z*	*U*_iso_*/*U*_eq_	Occ. (<1)
Cu1	0.12529 (5)	0.7500	1.11061 (15)	0.0314 (2)	
Br1	−0.10466 (3)	0.66291 (3)	0.15521 (10)	0.0450 (2)	
O1	0.1647 (2)	0.68206 (19)	1.3271 (6)	0.0423 (9)	
O2	0.2119 (3)	0.7500	0.8053 (11)	0.0547 (10)	
H2A	0.1990	0.7500	0.6587	0.066*	
N1	0.0729 (2)	0.6827 (2)	0.9116 (7)	0.0301 (9)	
C1	0.0321 (3)	0.7138 (3)	0.7227 (9)	0.0347 (9)	
C2	−0.0080 (3)	0.6782 (3)	0.5479 (9)	0.0367 (9)	
H2	−0.0076	0.6304	0.5456	0.044*	
C3	−0.0485 (3)	0.7142 (3)	0.3777 (9)	0.0327 (10)	
C4	0.0738 (3)	0.6172 (3)	0.9476 (10)	0.0394 (8)	
H4	0.0472	0.5905	0.8391	0.047*	
C5	0.1119 (3)	0.5817 (3)	1.1386 (9)	0.0392 (8)	
C6	0.1546 (3)	0.6165 (3)	1.3181 (9)	0.0397 (9)	
C7	0.1889 (3)	0.5735 (3)	1.4984 (10)	0.0416 (9)	
H7	0.2177	0.5936	1.6182	0.050*	
C8	0.1056 (3)	0.5104 (3)	1.1487 (10)	0.0425 (9)	
H8	0.0772	0.4889	1.0308	0.051*	
C9	0.1388 (3)	0.4700 (3)	1.3222 (10)	0.0441 (10)	
H9	0.1339	0.4224	1.3226	0.053*	
C10	0.1804 (3)	0.5040 (3)	1.4990 (11)	0.0433 (10)	
H10	0.2030	0.4779	1.6207	0.052*	
C11	0.2775 (5)	0.7500	0.8417 (18)	0.067 (2)	
H11A	0.3003	0.7274	0.7040	0.101*	0.50
H11B	0.2878	0.7260	0.9947	0.101*	0.50
H11C	0.2938	0.7966	0.8539	0.101*	0.50

### Atomic displacement parameters (Å^2^)

**Table:**

	*U*^11^	*U*^22^	*U*^33^	*U*^12^	*U*^13^	*U*^23^
Cu1	0.0344 (5)	0.0327 (5)	0.0269 (4)	0.000	−0.0054 (4)	0.000
Br1	0.0519 (4)	0.0464 (4)	0.0369 (3)	−0.0063 (3)	−0.0153 (2)	−0.0044 (2)
O1	0.053 (2)	0.039 (2)	0.036 (2)	0.0042 (18)	−0.0152 (17)	0.0006 (16)
O2	0.048 (2)	0.076 (2)	0.040 (2)	0.000	−0.0030 (18)	0.000
N1	0.031 (2)	0.032 (2)	0.027 (2)	0.0034 (18)	−0.0029 (17)	0.0005 (18)
C1	0.0348 (17)	0.0422 (17)	0.0272 (16)	0.0003 (15)	−0.0009 (15)	0.0008 (15)
C2	0.0377 (19)	0.042 (2)	0.0308 (18)	−0.0010 (17)	−0.0037 (17)	0.0007 (17)
C3	0.033 (2)	0.040 (2)	0.0252 (19)	−0.0030 (18)	−0.0033 (17)	−0.0008 (18)
C4	0.0398 (16)	0.0449 (17)	0.0336 (16)	0.0007 (15)	−0.0047 (14)	0.0008 (14)
C5	0.0406 (17)	0.0443 (17)	0.0327 (16)	0.0021 (15)	−0.0034 (15)	0.0009 (15)
C6	0.0397 (17)	0.0469 (18)	0.0326 (16)	0.0026 (16)	−0.0015 (15)	0.0020 (15)
C7	0.0446 (19)	0.0446 (19)	0.0355 (18)	0.0035 (18)	−0.0059 (17)	0.0017 (17)
C8	0.0452 (18)	0.0444 (18)	0.0380 (18)	0.0005 (17)	−0.0052 (16)	0.0017 (16)
C9	0.048 (2)	0.044 (2)	0.0400 (19)	0.0020 (18)	−0.0039 (17)	0.0039 (17)
C10	0.047 (2)	0.0451 (19)	0.0381 (19)	0.0044 (18)	−0.0044 (17)	0.0055 (17)
C11	0.052 (4)	0.090 (4)	0.060 (4)	0.000	0.002 (4)	0.000

### Geometric parameters (Å, °)

**Table:**

Cu1—O1^i^	1.908 (4)	C4—C5	1.430 (7)
Cu1—O1	1.908 (3)	C4—H4	0.9300
Cu1—N1^i^	1.960 (4)	C5—C8	1.390 (8)
Cu1—N1	1.960 (4)	C5—C6	1.429 (7)
Cu1—O2	2.324 (6)	C6—C7	1.432 (7)
Br1—C3	1.886 (5)	C7—C10	1.358 (8)
O1—C6	1.289 (7)	C7—H7	0.9300
O2—C11	1.271 (9)	C8—C9	1.368 (7)
O2—H2A	0.8199	C8—H8	0.9300
N1—C4	1.285 (7)	C9—C10	1.399 (8)
N1—C1	1.410 (6)	C9—H9	0.9300
C1—C2	1.392 (7)	C10—H10	0.9300
C1—C1^i^	1.405 (10)	C11—H11A	0.9600
C2—C3	1.383 (7)	C11—H11B	0.9600
C2—H2	0.9300	C11—H11C	0.9600
C3—C3^i^	1.388 (10)		
			
O1^i^—Cu1—O1	87.5 (2)	N1—C4—H4	116.9
O1^i^—Cu1—N1^i^	93.95 (16)	C5—C4—H4	116.9
O1—Cu1—N1^i^	172.22 (18)	C8—C5—C6	119.6 (5)
O1^i^—Cu1—N1	172.22 (18)	C8—C5—C4	117.7 (5)
O1—Cu1—N1	93.95 (16)	C6—C5—C4	122.7 (5)
N1^i^—Cu1—N1	83.6 (2)	O1—C6—C5	125.3 (5)
O1^i^—Cu1—O2	98.09 (16)	O1—C6—C7	118.9 (5)
O1—Cu1—O2	98.09 (16)	C5—C6—C7	115.9 (5)
N1^i^—Cu1—O2	89.29 (16)	C10—C7—C6	121.7 (5)
N1—Cu1—O2	89.29 (16)	C10—C7—H7	119.1
C6—O1—Cu1	127.0 (3)	C6—C7—H7	119.1
C11—O2—Cu1	126.8 (6)	C9—C8—C5	123.8 (5)
C11—O2—H2A	116.4	C9—C8—H8	118.1
Cu1—O2—H2A	116.8	C5—C8—H8	118.1
C4—N1—C1	122.6 (4)	C8—C9—C10	116.7 (6)
C4—N1—Cu1	124.8 (3)	C8—C9—H9	121.6
C1—N1—Cu1	112.6 (3)	C10—C9—H9	121.6
C2—C1—C1^i^	119.8 (3)	C7—C10—C9	122.3 (5)
C2—C1—N1	124.8 (5)	C7—C10—H10	119.2
C1^i^—C1—N1	115.4 (3)	C9—C10—H10	118.5
C3—C2—C1	119.9 (5)	O2—C11—H11A	109.5
C3—C2—H2	120.1	O2—C11—H11B	109.5
C1—C2—H2	120.1	H11A—C11—H11B	109.5
C2—C3—C3^i^	120.3 (3)	O2—C11—H11C	109.5
C2—C3—Br1	117.7 (4)	H11A—C11—H11C	109.5
C3^i^—C3—Br1	121.90 (15)	H11B—C11—H11C	109.5
N1—C4—C5	126.3 (5)		

Symmetry codes: (i) *x*, −*y*+3/2, *z*.

### Hydrogen-bond geometry (Å, °)

**Table:**

*D*—H···*A*	*D*—H	H···*A*	*D*···*A*	*D*—H···*A*
O2—H2A···O1^ii^	0.82	2.30	3.009 (6)	145

Symmetry codes: (ii) *x*, *y*, *z*−1.
